# Patient Panel Handoffs for New Interns in Internal Medicine Residency Continuity Clinics

**DOI:** 10.7759/cureus.71894

**Published:** 2024-10-19

**Authors:** Anna L Golob, Ginger A Evans, Whitney Harper, Christopher J Wong

**Affiliations:** 1 Division of General Internal Medicine, Department of Medicine, University of Washington, Seattle, USA; 2 Career Services, Colgate University, Hamilton, USA

**Keywords:** continuity clinic, graduate medical education, handoffs, internal medicine residency training, primary care

## Abstract

Background: Compared to inpatient care transitions, end-of-year resident continuity clinic panel transitions affect a greater number of patients, yet warm handoffs occur less often.

Objective: We developed a program-wide curriculum to implement warm handoffs (defined as in-person or virtual via videoconference) for high-risk continuity clinic patients between graduating and incoming residents.

Methods: The warm handoff intervention was phased in at different clinic sites over the study period and ultimately implemented program-wide across nine affiliated continuity clinics. Graduating residents were instructed to identify high-risk panel patients and optimize documentation of key patient care information for handoff. They then participated in a structured, in-person warm handoff event in June during intern orientation involving a direct transfer of information to incoming interns. We surveyed residents between 2017 and 2021 to assess their satisfaction with the continuity clinic handoff process, as well as their perceptions about safety outcomes, comparing those who received a warm handoff to those who did not.

Results: Achieving warm handoffs was feasible, reported by 72% (23/32) of intern respondents by the end of the study period, compared to 43% (13/30) during the first year. Residents who received a warm handoff were more likely to prefer warm handoffs (adjusted odds ratio (aOR) 8.1) and to report satisfaction with the handoff process (aOR 2.7). They were less likely to report having near-misses or adverse events. There were no statistically significant differences in attitudes regarding the importance of outpatient handoffs.

Conclusion: Structured warm handoffs of high-risk resident continuity clinic patients from graduating senior residents to incoming interns are feasible and associated with improved resident satisfaction with the continuity clinic panel transfer process and fewer perceived adverse patient care events during this vulnerable time of transition.

## Introduction

Each year, the graduation of residents who practice in continuity teaching clinics leads to a large, en masse transition in primary care physicians. Over 12,000 internal medicine (IM) and family medicine residents graduate each year [1,2]. Assuming a primary care panel size of approximately 100 patients per resident, over a million people cared for in teaching clinics are estimated to change physicians each July. These year-end resident continuity clinic panel transitions create a risk for adverse patient outcomes [3]. Moreover, as academic medical centers provide a disproportionate amount of care for the underserved [4], this transition period between primary care physicians renders potential gaps in care for vulnerable patient populations.

Numerous studies have addressed inpatient handoffs [5], while far fewer have focused on outpatient handoffs. Previous studies have demonstrated that an enhanced year-end handoff process from departing third-year residents (R3s) to rising second-year residents (R2s) improved care for a selected group of high-risk patients [3,6,7], and a structured sign-out using a task list between graduating R3s and incoming first-year residents (R1s) increased completion of those tasks [8].

Despite this evidence, only 34% of IM residency programs report having a year-end outpatient handoff system, and only 4% report assessing competency in these skills [9]. A smaller study of 12 IM residency programs found that, while all had a panel transfer process, only two of 12 had a verbal handoff system [10].

We implemented a structured, face-to-face warm handoff process for high-risk continuity clinic panel patients from graduating senior residents to incoming interns in a large, multi-site, university-based residency program. We conducted a retrospective evaluation of resident attitudes, perceptions, and satisfaction with the handoff process.

## Materials and methods

Intervention: At the University of Washington Internal Medicine Residency Program, a warm handoff evening event was initially piloted at a single clinic site starting in 2014. A second large clinic site joined the pilot in 2017. Starting in 2020, the handoff intervention was expanded to all program-affiliated continuity clinic sites (n=9 unique clinics) and designed as a half-day event during intern (R1) orientation.

During the final three months of residency training, R3s were instructed to identify high-risk panel patients and optimize electronic health record documentation of key patient care information for the handoff. To identify high-risk patients, graduating residents were encouraged to use available risk assessment tools such as the “Comprehensive Assessment of Needs” (CAN) [11] at the Veterans Affairs clinic or the electronic health record risk tool for those clinics using the Epic system (Epic Systems, Verona, WI), as well as their own judgment based on their knowledge of their patients. Documentation and communication tips for handoffs were modeled after previously published tools [12].

To begin the warm handoff event, participating residents first attended a didactic session addressing the rationale for continuity clinic handoffs, as well as transition skills for meeting a continuity clinic patient for the first time. Graduating R3s and incoming R1s then met face-to-face (in person or virtually) at each clinic to discuss specific high-risk patients. The handoff included a review of to-do items, as well as important social and contextual patient history. Any notes were kept at the clinics or saved securely within the electronic health record (EHR) to protect patient health information. Residents who could not attend the warm handoff event were asked to meet separately or by telephone at an alternate time, or if not feasible, to study panel patient charts for handoff information. 

Data collection: Residents are anonymously surveyed by the program twice yearly on a variety of topics pertaining to the continuity clinic educational experience for quality improvement purposes. Between 2017 and 2022, surveys included questions concerning continuity clinic panel handoffs (including R1 start dates from June 2017 to June 2021).

Data analysis: As this was not a controlled intervention, data were analyzed retrospectively, comparing those who reported receiving a warm handoff (in-person or video) to those who did not. Since the intervention took place in June, the primary analysis focused on survey responses from R1s that same academic year (distributed in December or January) (n=161). For the primary analysis, R2 and R3 survey responses were excluded, as they may have already responded as an R1 in a previous survey. Likert variables for agreement were dichotomized into binary variables with positive indicating whether the resident expressed agreement and negative indicating neutrality or disagreement. Frequency variables were also dichotomized with positive assigned to occurring “some” of the time or greater. Odds ratios were calculated using logistic regression with adjustment for clinic site, year of survey, and program track (categorical or primary care). Statistical analyses were conducted using R Studio software, version 2024.04.2+764 (Posit Software, PBC).

The continuity clinic survey is voluntary and distributed to all residents of the internal medicine residency program. As this was a retrospective analysis, consent was not obtained at the time of survey completion. This analysis was determined to be non-research per the University of Washington Human Subjects Research Determination process and did not require additional human subjects' approval.

## Results

Feasibility of warm handoff: R1 respondents who reported receiving an in-person handoff increased over time from 43% (13/30) in 2017 to 72% (23/32) in 2021.

Attitudes and preferences: Results are summarized in Table 1. R1s who received a warm handoff were much more likely to prefer warm handoffs for the transition of high-risk continuity clinic patients (adjusted odds ratio (aOR) 8.1). There were no statistically significant differences in attitudes toward the importance of continuity clinic handoffs, timing of seeing high-risk patients, phone calls prior to the first visit, or the need for a more detailed handoff process.

**Table 1:** Association between receipt of warm handoff and R1 response to questions about attitudes, preferences, and clinical experience. * Likert scale answers were analyzed as binary outcomes. For the preferred handoff method, face-to-face was the positive outcome, with all other choices (e.g., email, chart documentation) considered negative. The agreement was considered positive if respondents selected the upper two choices on a five-point scale. Frequency outcomes were considered positive if the response was greater than Very Rarely or Rarely (i.e., the top 3 out of a five-point scale). ** Odds ratios are adjusted for clinic site, program track (primary care, categorical), and year of survey.

Question	Outcome*	Association of Outcome and Receipt of Warm Handoff					
		Unadjusted			Adjusted**		
		Odds Ratio	95% CI	P-value	Odds Ratio	95% CI	P-value
Attitudes and Preferences							
What is your preferred method of handoff for your high-risk continuity clinic patients?	Warm handoff vs other	11.89	5.04, 28.01	<0.001	8.09	2.73, 23.98	< 0.001
Ambulatory hand-offs are just as important as inpatient handoffs.	Agreement	1.18	0.56, 2.47	0.670	0.77	0.26, 2.29	0.636
Seeing high-risk patients in the clinic as soon as possible after provider transfer would improve patient care.	Agreement	1.15	0.52, 2.54	0.734	0.71	0.21, 2.36	0.576
A telephone call with a high-risk patient prior to our first visit would help me assume their care.	Agreement	1.14	0.61, 2.13	0.683	0.88	0.39, 1.99	0.755
A more detailed sign-out/hand-off process would improve patient safety.	Agreement	0.84	0.42, 1.69	0.626	0.83	0.34, 2.05	0.692
Clinical Experience							
I am satisfied with how my high-risk continuity patients were handed off to me.	Agreement	4.48	2.29, 8.75	< 0.001	2.68	1.13, 6.36	0.026
I needed guidance at the beginning of my intern year on how to identify high-risk clinic patients.	Agreement	1.85	0.90, 3.79	0.094	1.20	0.43, 3.29	0.730
I am comfortable responding to requests from patients whom I've never seen.	Agreement	2.56	1.10, 5.98	0.030	1.37	0.48, 3.93	0.560
How often have you discovered information about your continuity clinic patients that should have been discussed in a handoff?	Sometimes or more frequently	0.70	0.38, 1.32	0.274	0.77	0.33, 1.77	0.535
How often have adverse events or near-misses occurred due to inadequate handoffs?	Sometimes or more frequently	0.06	0.01, 0.48	0.008	0.06	0.00, 0.96	0.047

Clinical experience: R1s who received a warm handoff were more likely to report satisfaction with how their high-risk continuity clinic patients were signed out to them compared to those who did not receive a warm handoff (aOR 2.7). They also were less likely to report having had near-misses or adverse events (aOR 0.06). There were no statistically significant differences for the following: whether they felt they needed guidance at the beginning of the year to identify high-risk patients, whether they felt comfortable responding to requests from patients they had not seen yet, or whether they more than rarely found information about their patients that should have been discussed in a handoff (Table 1).

Additional analysis of all resident respondents (R1s, R2s, R3s; total n = 433) found that the preference for warm handoffs and satisfaction with the handoff process remained associated with having received a warm handoff (aOR 7.5 (95% CI: 4.2-13.4) and 3.4 (95% CI: 2.1-5.7), respectively). In this expanded cohort, residents who received a warm handoff were less likely to report discovering information that should have been discussed in a handoff (aOR 0.60, 95% CI: 0.36-0.98) or finding adverse events or near-misses due to inadequate handoffs (aOR 0.45, 95% CI: 0.22-0.94).

## Discussion

Since 2016, there have been formal recommendations to implement verbal handoffs for high-risk outpatients [13], yet uptake has been limited [10]. This study demonstrates that a warm handoff process of high-risk continuity clinic panel patients cared for by internal medicine residents is feasible and associated with increased satisfaction in the handoff process, a preference for warm handoffs as the method of sign-out, and lower reporting of experiencing near-misses or adverse events.

Feasibility can be a challenge when creating a clinic patient handoff process. There is a limited time window when a graduating senior resident may overlap with an incoming intern. In some cases, a senior resident may have left the geographic area before the incoming intern arrives. Asking residents to hand off patients to each other on their own time risks competing with other demands. Despite these barriers, our study found that, with program sponsorship of the handoff process, and inclusion with intern orientation activities, a program-wide warm handoff is feasible. While previous studies have focused on either sign-out to a rising second-year resident [6], or more passive, asynchronous methods such as task lists [8], our study demonstrates that continuity clinic handoff can be accomplished on a larger scale, face-to-face, and with the more typical graduating R3 to incoming R1 panel transition.

Intern satisfaction with the transition of care for their high-risk patients was strongly associated with having received a warm handoff. Starting a primary care clinic can be a daunting journey for internal medicine residents, with nearly half feeling unprepared at the start of residency [14]. This study adds to the literature on ways to systematically improve the outpatient training experience for internal medicine residents. Receipt of a warm handoff alone, however, was not associated with comfort in fielding patient requests, suggesting that additional continuity clinic training is still required, consistent with other studies of interventions such as boot camps [15,16].

The finding that residents who received a warm handoff were less likely to report near-misses or adverse events should be interpreted with caution, as this study primarily describes the residents’ experience and was not designed to assess clinical outcomes. Interestingly, while the primary study population of interns did not report a difference in discovering information that should have been discussed in a handoff, in the expanded cohort, which included senior residents, having a warm handoff was associated with a lower likelihood. This discrepancy may be explained by the study design: halfway through the first year of residency, interns may not have seen all of their continuity clinic patients yet, and it is possible that, over the ensuing years, they discovered more information that they would have found helpful in a handoff.

There were no differences found between attitudes toward the importance of outpatient handoffs or the timing and method of seeing high-risk patients based on having received a warm handoff. This finding suggests that there is a more general understanding of the value of primary care for high-risk patients among internal medicine residents.

Limitations of this study include its single-site design, lack of randomization, and absence of clinical outcomes. Resident demographic data was intentionally not collected to retain anonymity among respondents, potentially limiting additional adjustment variables. Study strengths include the novel approach of holding the warm handoff event during intern orientation before graduating R3s have exited the program, implementing the handoff event across multiple heterogeneous continuity clinic sites, and having multi-year data including before and after program-wide implementation.

## Conclusions

This study demonstrates that a structured, face-to-face handoff process is both feasible and beneficial. Future directions could include evaluating the reproducibility of this intervention at other internal medicine training programs, as well as ways to capture the remaining residents who are not able to attend handoff events, such as increased use of asynchronous video conferencing.
